# Selective Influences of Maximum Dynamic Strength and Bar-Power Output on Team Sports Performance: A Comprehensive Study of Four Different Disciplines

**DOI:** 10.3389/fphys.2018.01820

**Published:** 2018-12-17

**Authors:** Irineu Loturco, Timothy Suchomel, Lachlan P. James, Chris Bishop, César C. C. Abad, Lucas A. Pereira, Michael R. McGuigan

**Affiliations:** ^1^NAR – Nucleus of High Performance in Sport, São Paulo, Brazil; ^2^Department of Human Movement Sciences, Carroll University, Waukesha, WI, United States; ^3^La Trobe Sport and Exercise Medicine Research Centre, Department of Rehabilitation, Nutrition and Sport, School of Allied Health, La Trobe University, Melbourne, VIC, Australia; ^4^Faculty of Science and Technology, London Sports Institute, Middlesex University, London, United Kingdom; ^5^Sports Performance Research Institute New Zealand, Auckland University of Technology, Auckland, New Zealand; ^6^School of Medical and Health Sciences, Edith Cowan University, Perth, WA, Australia

**Keywords:** muscle power, optimal loads, straight speed, sprinting, explosiveness

## Abstract

This study examined the selective influences of one-repetition maximum (1RM) values [assessed in the half-squat (HS)] and bar-power production [assessed in both HS and jump squat (JS) exercises] on the physical performance of male and female team sport athletes from four different sports. Three-hundred and three elite players (31 Olympians) from four different disciplines (47 male soccer players, 58 female soccer players, 28 male handball players, 58 female handball players, 49 male rugby players, and 63 male futsal players) participated in this study. The physical tests were performed over 2 consecutive days for soccer and rugby players, and in 1 day for the remaining athletes. On the first day, rugby and soccer athletes performed squat jumps (SJ), countermovement jumps (CMJ), and HS 1RM. On the second day, they executed HS and JS tests (to assess the maximum bar-power output) and the linear and change-of-direction (COD) speed tests. For the other players, the sequence of the measurements was the same; however, they did not perform the HS exercise. Athletes were separated, using a median split analysis, into two distinct groups, according to their bar-power output in both JS and HS exercises and their performance in HS 1RM. The magnitude-based inferences method was used to examine the differences between “higher” and “lower” performance groups. Overall, the bar-power outputs were better connected to improved acceleration, speed, and jump performance than the 1RM measures. From these findings, it is possible to infer that players able to produce higher bar-power outputs are likely to sprint faster and jump higher. Therefore, coaches involved in team sports are strongly encouraged to use the bar-power method to evaluate the athletic performance of their players.

## Introduction

Strength and power capabilities play a key role in team sports performance. Several studies have shown that stronger and more powerful players of different sports are usually capable of accelerating faster, jumping higher, and changing direction more rapidly (Wilson et al., 1993; McBride et al., 2005; Newton et al., 2006; Loturco et al., 2016b; Freitas et al., 2018). In addition, research has indicated that even more specific sport tasks such as throwing, kicking, and tackling seem to be positively influenced by the individual ability to generate greater levels of force and power (Marques et al., 2007; Kilduff et al., 2008; Loturco et al., 2014, 2016a; Dello Iacono and Seitz, 2018; Loturco et al., 2018a). Therefore, coaches and sport scientists are constantly seeking better and more accurate methods to properly improve and assess neuromuscular function in top-level athletes.

The one-repetition maximum (1RM) test is one of the most widely used measurements in the field of sport science (Fleck, 1999; Channell and Barfield, 2008; Suchomel et al., 2016a). Through this test, coaches can determine the maximum load that a subject can move during a maximum-effort resistance exercise (Loturco et al., 2018c) and thereby prescribe relative loads [i.e., 1RM percentages (% 1RM)], according to the athlete’s needs and objectives (e.g., strength or power development) (McMaster et al., 2013). Many studies have reported the effectiveness of 1RM-based training programs in improving the physical performance of team sport athletes. For example, Bogdanis et al. (2009) found significant increases in maximum and relative half-squat (HS) strength, change-of-direction (COD), vertical jumping, and sprinting abilities in senior soccer players who performed 6 weeks of HS training using loads ranging from 70 to 90% 1RM. Similarly, Appleby et al. (2012) showed that a long-term periodized training model with loads from 60 to 100% 1RM resulted in significant increases in body mass (BM), lean mass index, and upper-body strength in professional rugby union players. However, despite their popularity, some authors have raised concerns over the safety and usability of 1RM tests in professional sport settings (Chapman et al., 1998; Brown and Weir, 2001; Loturco et al., 2015d), where athletes regularly perform various concurrent and complementary activities, and time and resources are inherently limited (Bishop, 2008; Bishop et al., 2017; Freitas et al., 2018).

These issues are even more pronounced in large groups of individuals, which greatly compromise the use of 1RM measurements in team sport disciplines (Loturco et al., 2015d). To minimize these possible drawbacks and optimize performance gains, we proposed the use of an alternative training and testing strategy, based on barbell power production (Loturco et al., 2018c). In this regard, instead of considering only the “maximum mass” moved in a given exercise, the “bar-power approach” reflects, at the same time, the force and velocity applied to the barbell (Loturco, 2017; Loturco et al., 2018c). With this method, practitioners can safely determine the loads capable of maximizing bar-power output, using rapid incremental loading tests or instantaneously measuring the optimum bar-velocities (Loturco et al., 2015b, 2017d). To date, although a number of studies have confirmed the efficiency of the optimum power loads (OPL) to improve the physical performance of team sport athletes, these investigations were executed with male players of specific sport disciplines (e.g., soccer and basketball) (Loturco et al., 2017a; Dello Iacono and Seitz, 2018; Freitas et al., 2018). Knowing more about the relationships between bar-power output and the athletic abilities of both male and female athletes of different sports may lead researchers to develop new studies regarding this topic, as well as stimulate coaches to implement this strategy in their professional practices. Moreover, the possibility of comparing the magnitude of these correlations with those related to more traditional performance measures (e.g., 1RM values) could also reinforce and support the use of the OPL in high performance sport.

As such, a recent study using a pooled sample of 61 elite athletes from four different sports (i.e., track and field, rugby sevens, soccer, and bobsled) compared these mechanical relationships, revealing that the bar-power outputs are more strongly associated with linear speed and vertical jump height than 1RM values (Loturco et al., 2018c). Nevertheless, a more comprehensive investigation is warranted by reporting these data in a more specific way (i.e., with subjects grouped on a sport-by-sport basis), involving male and female players of different field and court team sports (e.g., handball and futsal) and with additional performance outcomes (e.g., COD speed). An alternative strategy for estimating the influence of a given exercise on performance is examining the data provided by the median split analysis (Rampinini et al., 2007; Iacobucci et al., 2015). Based on this method, practitioners can group the athletes according to their physical skills, defining the lower and upper bounds of performances in a series of assessments. Under this rationale, it seems plausible to consider that superior levels of performance in two or more measurements might be closely interconnected, representing shared and direct relations between them (Loturco et al., 2017f).

Thus, the aim of this study was to test and compare the interconnection between bar-power output [collected in the HS and jump squat (JS) exercises] and 1RM values (collected in HS) and a variety of sport-specific performance measures (i.e., linear speed, COD, acceleration and jump abilities) in male and female elite players of four different sports (rugby, soccer, futsal, and handball).

## Materials and Methods

### Participants

Three-hundred and three elite athletes (47 male soccer players, 58 female soccer players, 28 male handball players, 58 female handball players, 49 male rugby players, and 63 male futsal players) from four different sports participated in this study. The characteristics of the subjects are presented in Table 1. Male soccer players participated in the first division of the Paulista State Championship. Female soccer players participated in the first division of the Brazilian National Championship and won the 2017 Libertadores da America Cup. Male and female handball players participated in the first division of the Brazilian National Championships, comprising 39 (15 male and 24 female) athletes of the Brazilian National Team, and 23 (11 male and 12 female) who participated at the Rio-2016 Olympic Games. Rugby players were members of the Brazilian National Team comprising nine athletes who participated in the rugby sevens tournament at the Rio-2016 Olympic Games. Finally, futsal players won the 2016 Brazilian National League. Therefore, we can confirm the high level of performance of the participants in this study. This study was carried out in accordance with the recommendations of the Anhanguera-Bandeirante Ethics Committee with written informed consent from all subjects. All subjects gave written informed consent in accordance with the Declaration of Helsinki. The protocol was approved by the Anhanguera-Bandeirante Ethics Committee.

**Table 1:** Characteristics of the subjects (mean ± standard deviation) of the four different sports disciplines.

	Male Soccer	Female Soccer	Male Handball	Female Handball	Rugby	Futsal
Age (years)	22.5 ± 2.9	22.6 ± 7.6	28.3 ± 3.2	25.2 ± 4.3	24.4 ± 4.2	23.5 ± 3.3
Weight (kg)	71.2 ± 8.8	61.0 ± 7.6	90.3 ± 10.3	69.7 ± 7.3	88.8 ± 10.0	73.6 ± 6.9
Height (cm)	177.1 ± 7.6	166.4 ± 6.9	188.3 ± 4.6	173.4 ± 5.8	179.1 ± 6.1	176.3 ± 5.7

### Study Design

The athletes involved in this study were assessed during the competitive phase of the season and were well familiarized with testing procedures due to their constant assessments in our facilities. Physical tests were performed on 2 consecutive days for soccer and rugby athletes and 1 day for the other athletes. For rugby and soccer players, on day 1, squat jumps (SJ), countermovement jumps (CMJ), and a 1RM HS were performed. Meanwhile, on day 2, the maximum bar-power outputs in the HS and JS exercises, and linear and COD sprint tests were assessed. For the other sports players the sequence of tests was the same, but they did not perform the 1RM test or the assessment of bar-power outputs in the HS exercise. Participants were required to be in a fasting state for at least 2 h, avoiding caffeine and alcohol consumption for 24 h before the procedures. Prior to the tests, the athletes performed standardized warm-up protocols including general (i.e., running at a moderate pace for 10-min followed by active lower limb stretching for 3-min) and specific workouts (i.e., submaximal attempts at each tested exercise). Between each test, a 15-min rest interval was allowed, to explain the procedures and adjust the equipment.

### Vertical Jumps

Vertical jump height was assessed using the SJ and CMJ. In the SJ, athletes were required to remain in a static position with a 90° knee flexion angle for ∼2-s before jumping, without any preparatory movement. In the CMJ, athletes were instructed to execute a downward movement followed by complete extension of the legs and were free to determine the countermovement amplitude to avoid changes in jumping coordination. All jumps were executed with the hands on the hips and the athletes were instructed to jump as high as possible. The jumps were performed on a contact platform (Elite Jump^®^, S2 Sports, São Paulo, Brazil) that has previously been shown to be valid and reliable (Loturco et al., 2017e). A total of five attempts were allowed for each jump, interspersed by 15-s intervals (Loturco et al., 2017e). The best attempts for the SJ and CMJ were used for the analyses.

### Maximum Dynamic Strength Test in the Half-Squat Exercise

Maximum dynamic strength was assessed using the 1RM HS test as described previously (Brown and Weir, 2001). Prior to the test, subjects executed two warm-up sets, as follows: (1) five repetitions at 50% of the estimated 1RM and; (2) three repetitions at 70% of the estimated 1RM. A 3-min rest interval was provided between all sets. After 3 min, athletes started the test and were allowed up to five attempts to achieve their 1RM (i.e., maximum weight that could be lifted once using proper technique), which was measured to the nearest 1 kg (Brown and Weir, 2001). The test was performed using Smith-machine equipment (Hammer-Strength Equipment, Rosemont, IL, United States). Values were normalized by dividing the 1RM by the athletes’ BM (i.e., relative strength = kg kg^-1^).

### Bar-Power Outputs in Jump Squat and Half-Squat Exercises

Maximum bar-power outputs were assessed in JS and HS, all performed on a Smith machine (Hammer Strength Equipment, Rosemont, IL, United States). Participants were instructed to execute three repetitions at maximal velocity for each load, starting at 40% of their BM in both exercises. In the JS, participants executed knee flexion until the thigh was parallel to the ground and, after the command to start, jumped as fast as possible without their shoulders losing contact with the bar. The HS was executed in a similar fashion to the JS, except that the subjects were instructed to move the bar as fast as possible without losing foot contact with the ground, keeping their heels on the floor. In both exercises, a load of 10% of BM was progressively added for each set until a clear decrement in mean power (MP), mean propulsive power (MPP), and peak power (PP) was observed (Loturco et al., 2018b). A 5-min rest period occurred between sets. To determine the power outputs, a linear position transducer (T-Force, Dynamic Measurement System; Ergotech Consulting S.L., Murcia, Spain) was attached to the Smith machine bar and values were automatically derived by the custom-designed software as follows: MP-value calculated during the entire concentric phase of each repetition; MPP – value calculated during the propulsive phase, defined as that portion of the concentric action during which the measured acceleration is greater than acceleration due to gravity; PP – the highest bar-power value registered at a particular instant (1-ms) during the concentric phase (Sanchez-Medina et al., 2010, 2014). The bar position data were sampled at 1000 Hz. The maximum MP, MPP, and PP values obtained in each exercise were used for analysis. Values were normalized by dividing the absolute power by the athletes’ BM (i.e., relative power = W kg^-1^) to produce more consistent relationships with athletic performance and allow for comparison with previous research (Cronin and Hansen, 2005; Cormie et al., 2007, 2010; Loturco et al., 2018c).

### Linear Sprint Tests

For the sprint test, rugby players performed a 40-m sprint test, whereas the other athletes sprinted over a total distance of 20-m. Four pairs of photocells (Smart-Speed, Fusion Equipment, Brisbane, QLD, Australia) were positioned at distances of zero, 5-, 10-, and 20-m along the sprinting course, and two additional pairs were placed at 30- and 40-m to assess rugby players. Sprint velocity (VEL) was calculated as the distance traveled over a measured time interval. The acceleration (ACC) capacity in the different distances (i.e., 0–5-, 5–10-, 10–20-, 20–30-, and 30–40-m) was calculated as the rate of change of velocity with respect to time. Athletes performed two sprints, interspaced by a 5-min rest interval, and the best attempt was retained for analysis.

### Zig-Zag Change of Direction Speed Test

**FIGURE 1:**
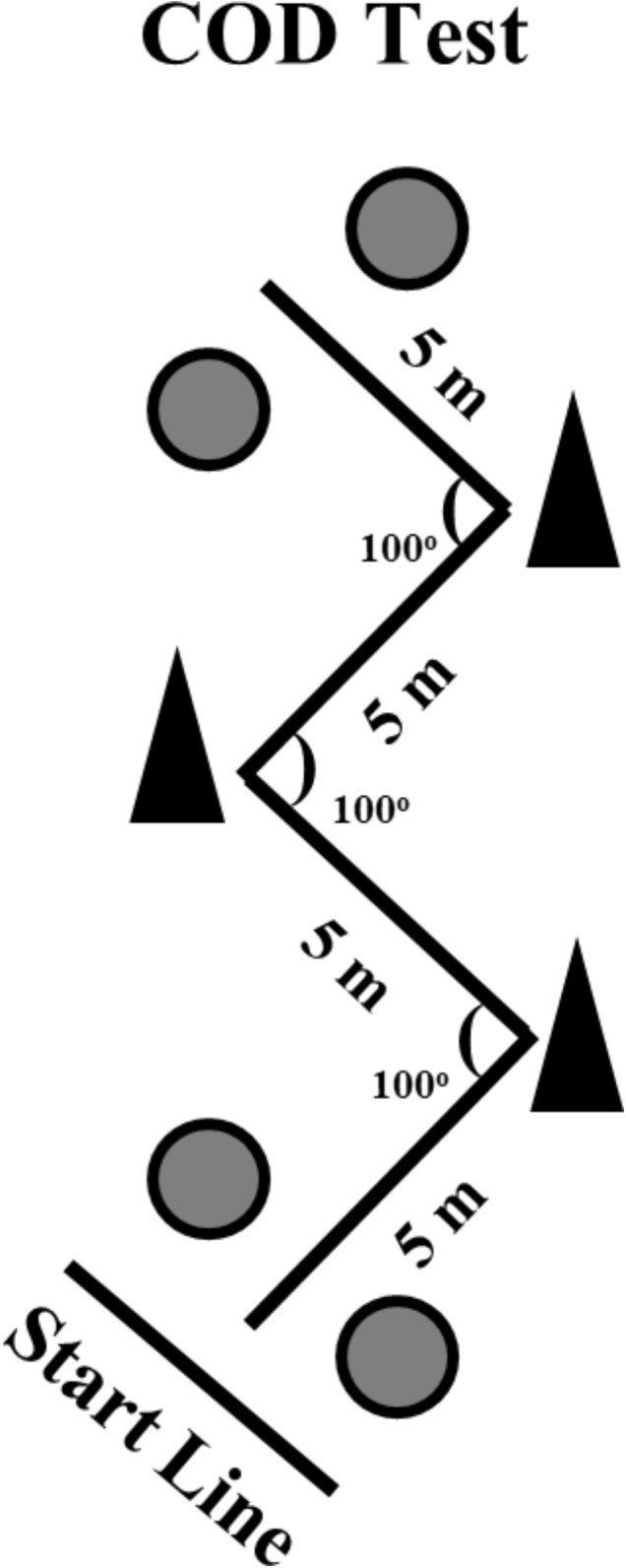
Schematic presentation of the change of direction speed test. The circles represent the positions of the photocells.

The Zig-zag COD test was performed on an indoor court and consisted of four 5-m sections (total 20-m of linear distance) marked with cones set at 100° angles (Figure 1) requiring the athletes to decelerate and accelerate as fast as possible around each cone. Two maximal attempts were performed with a 5-min rest interval between attempts. Starting from a standing position with the front foot placed 0.3-m behind the first pair of timing gates (Smart Speed, Fusion Equipment, Brisbane, QLD, Australia) (i.e., starting line), the athletes were instructed to complete the test as quickly as possible, until crossing the second pair of timing gates, placed 20-m from the starting line (Loturco et al., 2016c; Pereira et al., 2018). The fastest time from the two attempts was retained for further analysis.

### Statistical Analyses

Data are presented as means ± standard deviation. Data normality was tested using the Shapiro–Wilk test. Athletes were divided, using a median split analysis, into two groups according to their bar-power outputs in both exercises and HS 1RM (e.g., higher and lower JS MP, higher and lower HS MP, and higher and lower HS 1RM). The magnitude-based inferences method was used to analyze the differences between groups in the physical performance tests (Batterham and Hopkins, 2006). The magnitudes of the differences in the different performance variables were expressed as standardized mean differences [Cohen’s *d*, effect size (ES)]. The smallest worthwhile change (SWC) was set by using the Cohen’s principles for a small ES (i.e., 0.2) for each variable tested (Hopkins et al., 2009). To analyze the differences between groups, terms such as possibly and unclear were used if the 90% confidence limits (CL) crossed one or both SWC boundaries, respectively. Otherwise, if the CL did not cross SWC boundaries, the effect was inferred as probably. Additionally, the magnitudes of the standardized differences were interpreted using the following thresholds: <0.2, 0.2–0.6, 0.6–1.2, 1.2–2.0, 2.0–4.0, and >4.0 for trivial, small, moderate, large, very large, and near perfect, respectively (Hopkins et al., 2009). The assessments used in this research presented good levels of absolute and relative reliability (CV < 5% and ICC > 0.90, for all tested variables) (Hopkins et al., 2009).

## Results

All data presented a normal distribution. Table 2 shows the descriptive data of the vertical jumps, bar-power outputs in both JS and HS exercises, and 1RM in the HS exercise for the athletes of the different modalities assessed. Table 3 demonstrates the results of the linear sprint and COD speed tests for the athletes of four different sports disciplines.

**Table 2:** Descriptive results of the vertical jumps, bar-power outputs, and one repetition maximum in the athletes of four different sports disciplines.

	Male Soccer	Female Soccer	Male Handball	Female Handball	Rugby	Futsal
SJ (cm)	39.68 ± 4.05	31.25 ± 4.37	37.75 ± 5.24	30.07 ± 4.37	40.76 ± 6.11	37.82 ± 7.10
CMJ (cm)	41.05 ± 4.74	31.81 ± 4.21	40.64 ± 6.53	30.86 ± 4.01	42.76 ± 6.14	38.50 ± 4.88
JS MP (W kg^-1^)	5.43 ± 0.83	5.10 ± 0.87	6.32 ± 1.33	5.31 ± 1.04	7.28 ± 1.34	6.44 ± 1.43
JS MPP (W kg^-1^)	8.08 ± 1.04	7.28 ± 1.25	8.62 ± 1.68	7.30 ± 1.42	10.40 ± 1.92	9.20 ± 2.04
JS PP (W kg^-1^)	17.34 ± 2.06	16.16 ± 2.77	19.05 ± 3.72	16.17 ± 3.15	23.55 ± 4.51	20.43 ± 4.53
HS MP (W kg^-1^)	5.39 ± 0.37	–	–	–	7.38 ± 1.49	–
HS MPP (W kg^-1^)	7.48 ± 0.86	–	–	–	9.46 ± 1.90	–
HS PP (W kg^-1^)	15.60 ± 2.00	–	–	–	20.81 ± 4.19	–
HS 1RM (kg kg^-1^)	1.82 ± 0.14	–	–	–	2.24 ± 0.30	–

*Note: SJ, squat jump; CMJ, countermovement jump; JS, jump squat; HS, half-squat; MP, mean power; MPP, mean propulsive power; PP, peak power; 1RM, one-repetition maximum*.

**Table 3:** Descriptive results of the speed tests in the different distances tested in the athletes of four different sports disciplines.

	Male Soccer	Female Soccer	Male Handball	Female Handball	Rugby	Futsal
VEL 5-m (ms^-1^)	4.86 ± 0.25	4.35 ± 0.66	4.89 ± 0.33	4.62 ± 0.26	5.01 ± 0.31	4.81 ± 0.25
VEL 10-m (ms^-1^)	5.75 ± 0.19	5.14 ± 0.78	5.73 ± 0.29	5.29 ± 0.24	5.78 ± 0.28	5.68 ± 0.19
VEL 20-m (ms^-1^)	6.79 ± 0.22	5.96 ± 0.90	6.63 ± 0.28	6.06 ± 0.28	6.77 ± 0.31	6.61 ± 0.22
VEL 30-m (ms^-1^)	–	–	–	–	7.30 ± 0.33	–
VEL 40-m (ms^-1^)	–	–	–	–	7.64 ± 0.35	–
Zig–zag (ms^-1^)	3.37 ± 0.11	3.29 ± 0.11	3.54 ± 0.19	3.38 ± 0.15	3.63 ± 0.16	3.52 ± 0.11
ACC 0–5-m (ms^-2^)	4.74 ± 0.50	3.96 ± 0.32	4.80 ± 0.67	4.29 ± 0.52	5.05 ± 0.63	4.64 ± 0.50
ACC 5–10-m (ms^-2^)	1.26 ± 0.22	1.03 ± 0.16	1.16 ± 0.21	0.84 ± 0.18	1.04 ± 0.16	1.22 ± 0.22
ACC 10–20-m (ms^-2^)	0.86 ± 0.09	0.61 ± 0.09	0.71 ± 0.11	0.55 ± 0.09	0.81 ± 0.10	0.74 ± 0.09
ACC 20–30-m (ms^-2^)	–	–	–	–	0.47 ± 0.07	–
ACC 30–40-m (ms^-2^)	–	–	–	–	0.30 ± 0.06	–

*Note: VEL, velocity; ACC, acceleration*.

**FIGURE 2 F2:**
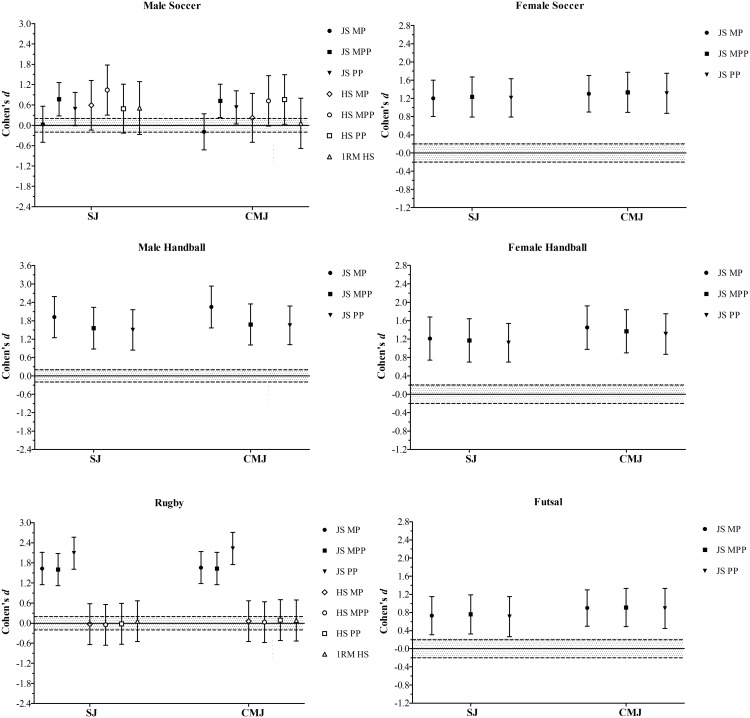
Standardized mean differences (Cohen’s *d*) of the squat and countermovement jumps (SJ and CMJ, respectively), comparing higher and lower groups divided by the bar-power outputs (MP, mean power; MPP, mean propulsive power; PP, peak power) in both jump squat (JS) and half-squat (HS) exercises, and one repetition maximum (1RM) in the HS exercise. The gray area represents the smallest worthwhile change (SWC) (0.20) based on Cohen’s principles for a small effect size; bar-errors represent 90% confidence limits (CL).

**FIGURE 3:**
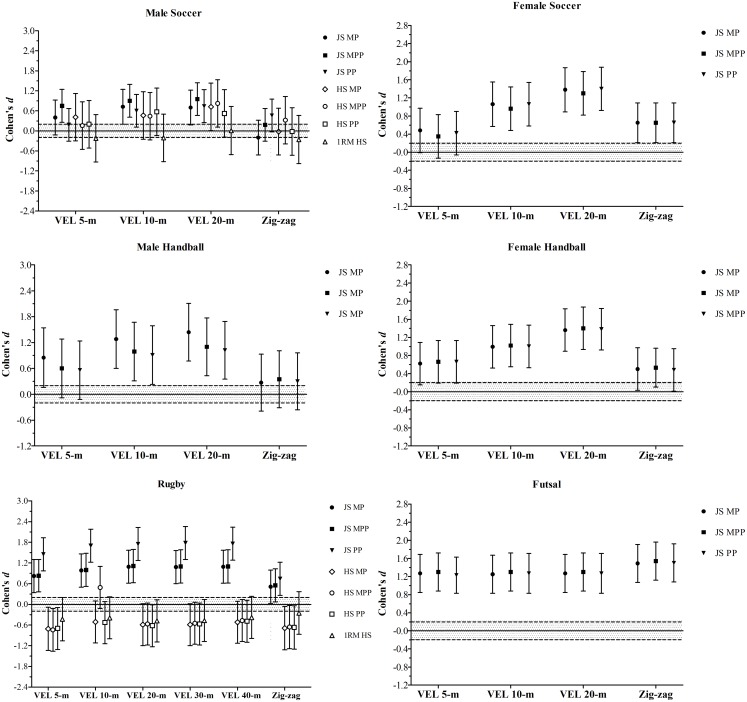
Standardized mean differences (Cohen’s *d*) of the sprint velocity (VEL) for the different distances tested and Zig-zag change of direction speed test, comparing higher and lower groups divided by the bar-power outputs (MP, mean power; MPP, mean propulsive power; PP, peak power) in both jump squat (JS) and half-squat (HS) exercises, and one repetition maximum (1RM) in the HS exercise. The gray area represents the SWC (0.20) based on Cohen’s principles for a small effect size; bar-errors represent 90% CL.

**FIGURE 4:**
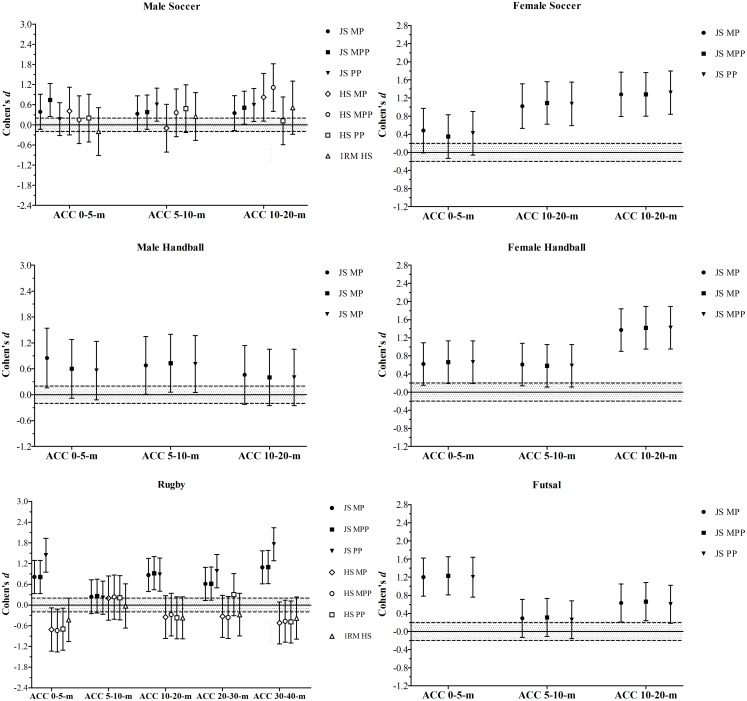
Standardized mean differences (Cohen’s *d*) of the sprint acceleration (ACC) for the different distances tested, comparing higher and lower groups divided by the bar-power outputs (MP, mean power; MPP, mean propulsive power; PP, peak power) in both jump squat (JS) and half-squat (HS) exercises, and one repetition maximum (1RM) in the HS exercise. The gray area represents the SWC (0.20) based on Cohen’s principles for a small effect size; bar-errors represent 90% CL.

Figure 2 shows the Cohen’s *d* for the comparisons between higher and lower 1RM and bar-power output groups in the SJ and CMJ height in the distinct groups of athletes. Figure 3 depicts the comparisons between higher and lower groups, divided based on their bar-power outputs and 1RM in the linear and COD speed tests in the athletes from the different sports disciplines. Figure 4 demonstrates the comparisons of the acceleration results comparing higher and lower bar-power outputs and 1RM groups in the distinct groups of athletes.

## Discussion

This study examined the selective influences of 1RM values (assessed in HS) and bar-power production (assessed in both HS and JS exercises) on the physical performance of male and female team sport athletes of four different sports (rugby, soccer, futsal, and handball). The main results reported here are: (1) overall, the bar-power outputs (i.e., MP, MPP, and PP) were more connected to better performances in speed and power assessments (i.e., jump, linear sprint, and COD tests) than the 1RM values, and (2) the players able to generate greater levels of bar-power were, in general, able to sprint faster, jump higher, and change direction more quickly than their less powerful peers. This is the first study to show this connection for the bar-power approach in a comprehensive sample of elite team sport athletes of different team sport disciplines.

A previous investigation using the same statistical approach (i.e., median split analysis) showed similar trends in National rugby players (Loturco et al., 2017f). However, the previous study did not compare the possible influences of bar-power outputs and 1RM measures on athletic performance. Even so, in line with the current findings, the results indicated that players capable of generating more power in the JS were equally capable of performing better in jump, COD, and sprint tests. In contrast, also in line with our data, higher performances in HS were not connected to superior performance in any functional assessment. As such, the novelty of including the 1RM measurement in this research was not able to increase the selective influence of HS exercise on athletic performance of elite rugby players. Although it is clear from the literature that the maximum dynamic strength plays a critical role in rugby performance (Argus et al., 2012; Comfort et al., 2012; McMaster et al., 2014), at least in these specific motor tasks (i.e., jump, acceleration, high-speed, and COD efforts), the HS 1RM measurement was not sensitive enough to differentiate national team rugby players with distinct physical performance levels. These results partially confirm and extend previous observations showing that: (1) the 1RM values are less related to sprint and jump performance than the power-related variables (Baker and Nance, 1999; Cunningham et al., 2013; Loturco et al., 2018c), and (2) the HS exercise seems not to be appropriate to predict or even monitor athletic performance in elite rugby players (Loturco et al., 2017f). Nonetheless, these data should be viewed with caution as previous research has suggested that enhanced force production (via the increased squat performance) might contribute to improved performance in professional rugby players (Comfort et al., 2012) Moreover, it has been reported that squat strength is strongly related to tackling ability in rugby league players (Speranza et al., 2015), an ability which was not measured in the current study. That said, in light of the above discussion, rugby practitioners are encouraged to include loaded JS assessments in their testing routines, especially when assessing elite rugby players.

As observed in rugby, higher or lower HS 1RM performances appeared to have no influence on jump, speed, and acceleration capabilities in male soccer players. In contrast to rugby athletes, the soccer players with higher HS bar-power outputs, overall, performed better than their weaker peers in all functional assessments (Figures [Fig F2]2, 3, and 4). These data contradict previous research showing strong correlations of maximal squat strength with sprint performance and vertical jump height in elite soccer players (Wisloff et al., 2004). To some extent, our results are similar to those of Requena et al. (2011), who found close relationships between traditional squat power output and sprint speed at 30- and 40-m. Although these authors also reported significant correlations between 1RM squat and sprint ability, only the power measures (i.e., maximal peak power and maximal average power) were significantly related to CMJ height (Requena et al., 2011). Nevertheless, in line with our findings, the associations between ballistic squats (i.e., loaded JS) and speed and jump variables were stronger than those detected for traditional squats. For many authors, the apparent superiority of JS over other resistance exercises to predict and improve athletic performance may be due to its kinematic and kinetic features (Baker, 1996; Cormie et al., 2011; Suchomel et al., 2016b; Loturco et al., 2017f). Accordingly, it has been shown that some “mechanical similarities” (Loturco et al., 2016c) between JS and certain speed-power tasks may positively affect the specific training adaptations, thus increasing the transference effect of JS bar-power outputs to performance. Overall, these observations support and reinforce the use of loaded JS to both evaluate and improve physical qualities in male soccer players.

Despite the absence of HS assessments in the following groups (precluding comparisons between HS and JS exercises), female soccer players, male futsal players, and male and female handball players with greater measures of bar power-output also perform better in both SJ and CMJ tests. These results are in accordance with those reported in several other studies and, as aforementioned, are likely related to the mechanical resemblances between loaded and unloaded vertical jumps (Figure 2) (Cronin and Hansen, 2005; Moir et al., 2005; McBride et al., 2010; Janssen et al., 2012; Loturco et al., 2015a, 2017b, 2018c). As a consequence, athletes able to generate higher levels of power during loaded JS (using light to moderate loads) can be expected to produce higher levels of power under unloaded jumping conditions (SJ and CMJ), and are also likely to jump higher (Loturco et al., 2015a). The same holds true for acceleration and speed capabilities (Figures 3 and 4), which have been shown to be strongly related to the JS maximum power output (Cronin and Hansen, 2005; Loturco et al., 2015a, 2017c). In fact, when a subject executes a loaded JS, they have to jump lifting up the whole mechanical system (i.e., weighted barbell + body mass), providing measurements automatically adjusted by the BM (Loturco et al., 2017c). Therefore, a greater JS performance might also indicate an increased ability to overcome the inertia and accelerate the body quickly and effectively, which is essential to achieve higher velocities over short distances (Cronin and Hansen, 2005; Loturco et al., 2015c; Kale and Acikada, 2016). Research by Cormie et al. (2011) supports this, stating that ballistic JS “circumvents any deceleration phase by requiring subjects to accelerate throughout the entire range of motion to the point of projection” (i.e., takeoff), being “more sport-specific for a vast number of sports.” Another advantage of using loaded JS to assess a number of strength-power variables is related to its high degree of reliability (Moir et al., 2005), achieved without the need to perform familiarization sessions, supporting the suitability of the tests for monitoring physical performance team sport athletes (who regularly perform many concurrent activities, within a congested schedule of engagements) (Loturco et al., 2015d; Freitas et al., 2018). Together, these data strongly support the notion that JS performed with a load that maximizes power output is one of the best methods to assess and improve physical performance in professional athletes from a wide variety of sports (Baker, 1996; Loturco et al., 2017c, 2018c).

A particular aspect of the current investigation is the lack of consistency in the outcomes related to COD performance across the examined sports (Figure 3). Briefly, COD speed can be characterized as a multifaceted ability, which relies on a series of different and multiple technical and physical aspects (e.g., stride adjustments, foot placement, straight speed, leg muscle qualities, etc.) (Young and Farrow, 2006; Brughelli et al., 2008; Hewit et al., 2012). This well-documented complexity could have affected the performance obtained by some athletes during the Zig-zag test (Little and Williams, 2005; Pereira et al., 2018), making this maneuver more convenient for assessing (for example) futsal players than male soccer players (Nimphius et al., 2010; Chaouachi et al., 2012). Indeed, previous research showed that the reduced pitch dimensions and more frequent turnovers during futsal match-play (compared to soccer), in both attacking and defending actions, support the development of higher coordinative skills in futsal players (Benvenuti et al., 2010). Although these differences were found in “reactive COD tasks,” these sport-related characteristics (and competences) may have influenced our results. However, these are only speculations and further work is needed to identify the most relevant factors for COD performance. Thus, this study confirms and strengthens previous conclusions, highlighting the necessity to create and adopt more effective training strategies to properly evaluate and develop COD ability in elite team sport athletes (Nimphius et al., 2010; Young and Farrow, 2006; Hewit et al., 2012).

In summary, this research shows that the bar-power approach is a useful method to assess team sport players, due to its close connection to acceleration, speed, and jumping abilities. These data are similar to those reported in a recent investigation, indicating that the bar-power outputs are more strongly associated with speed-power performances in elite athletes from four different sports than 1RM measurements (Loturco et al., 2018c). Therefore, as previously suggested, the possibility of using a range of loads which optimize the force and velocity applied to the barbell simultaneously [instead of only considering the maximum mass moved during a maximum effort (i.e., 1RM)] might better reflect the physical abilities and technical skills required in team-sport-tasks (Loturco, 2017; Loturco et al., 2018c). Finally, it is essential to emphasize that this work is inherently limited by its cross-sectional design, precluding inferences about causality. Nonetheless, our findings are strongly supported by a series of studies which has already demonstrated the effectiveness of the OPL (directly assessed on the barbell) to acutely or chronically improve performance in elite and sub-elite team sport athletes (Loturco et al., 2015d, 2016c; Dello Iacono and Seitz, 2018; Freitas et al., 2018).

## Conclusion

The bar-power approach is a practical and useful strategy to assess the physical performance of elite team sport players. Similar to previous findings (Loturco et al., 2018c), also in highly trained athletes, the bar-power output seems to be closely related to a series of athletic capabilities, which are recognized to play an important role in team sports performance, especially when considering the decisive game actions (Faude et al., 2012; Povoas et al., 2012; Ross et al., 2014). As described in other sport disciplines (Loturco et al., 2018c), it is likely that the opportunity to use measurements which consider, at the same time, the force and velocity applied to the barbell may have contributed to the stronger connections observed between bar-power variables and acceleration, speed, and jump qualities (when compared to the 1RM measures). Despite the lack of consistency and uniformity among the outcomes related to COD performance across the examined sports (which appears to be commonplace in COD studies) (Brughelli et al., 2008), it is possible to infer from these findings that players able to produce higher bar-power outputs are more prone to sprint faster and jump higher. From a general perspective, these “interconnections” are still more pronounced when the outcomes are directly collected from the loaded JS. Future studies should be conducted to test the causality between the variables reported here, as well as to search for more precise and consistent predictors of COD speed.

## Author Contributions

IL, LP, and MM: designed the work. IL, CA, and LP: data acquisition. IL, TS, LJ, CB, CA, LP, and MM: analysis and interpretation of data. IL and LP: drafting first version of the work. IL, TS, LJ, CB, CA, LP, and MM: critically revising the work. IL, TS, LJ, CB, CA, LP, and MM: final approval of the version to be published. IL, TS, LJ, CB, CA, LP, and MM: agreed to be accountable for all aspects of the work in ensuring that questions related to the accuracy or integrity of any part of the work were appropriately investigated and resolved.

## Conflict of Interest Statement

The authors declare that the research was conducted in the absence of any commercial or financial relationships that could be construed as a potential conflict of interest. The handling Editor declared a past co-authorship and collaboration with one of the authors MM.
